# Supplementary material for: The Art of the Consult Call: Improving Communication Through Shared Mental Models
Source: MedEdPORTAL. 2023 Sep 29;19:11347. doi: 10.15766/mep_2374-8265.11347 (PMC10539490; doi:10.15766/mep_2374-8265.11347)
Supplement: Supplementary file 1 — Session Overview.docxConsultation Cases 1 and 2.docxEvaluation and Consultation Components.docxDrawing Activity Materials.docxCurriculum Feedback Survey.docx [file mep_2374-8265.11347-s001.zip › A. Session Overview.docx]

**The Art of the Consult Call:**

**Improving Communication Through Shared Mental Models**

**Objectives:**

By the end of this session learners will be able to:

1. Summarize the elements of Kessler’s “5 Cs” of a consult call.
2. Connect the communication skills required for effective paired drawing to the elements of consultation.
3. Apply the elements of Kessler’s “5 Cs” in mock communication exercises.

**Set-Up/Materials:**

1. Markers/pens for each participant
2. Supplemental documents:
   1. Appendix B – Consultation Cases #1 and #2
   2. Appendix C – Evaluation and Consultation Components
   3. Appendix D – Drawing Activity Materials
   4. Appendix E – Curriculum Feedback Survey
3. Blank sheets of paper

**Clinical Case Activity #1 (in pairs): (5 min)**

1. Have participants pair off.  Designate the junior learner of the pair as Participant A and the other as Participant B.
2. Give Participant A the clinical case file. Instruct half the room to start with Case #1 and half the room to start with Case #2. Cases are available in Appendix B.
3. Give Participant B the scoring QR code or physical evaluation sheet, Appendix C.
4. Give all participants 30 seconds to look over their materials.
5. Participant A should perform a mock consult call where they are the ED physician calling a consulting service (Participant B). Participant B may ask clarifying questions but is not required to. This should take less than 2 minutes.
6. Participant B should complete the evaluation sheet and submit it electronically or in person.

**Artistic Activity #1 (in pairs): (5-10 min)**

1. Keep participants in their pairs. Each pair should now turn their chairs so they are sitting back-to-back and cannot see the paper that their partner is holding. Try to arrange so groups cannot clearly see the papers of other groups (very important!)
2. Give Participant B (the ‘giver’) the sheet with the house drawing. Give Participant A (the ‘receiver’) the mostly blank sheet with the boat prompt and a marker. Drawing activity is in Appendix D.
3. Have them follow the instructions on the sheet for 5-10 minutes.  The ‘giver’ can tell the ‘receiver’ which elements and strokes to make and where, but should not tell the ‘receiver’ what they are drawing (no “big picture” hints).

**Group Debrief & Discussion: (20 min)**

1. Have people share with the group and compare their drawings to the original drawing as well as other group’s drawing.
2. Facilitate discussion regarding the exercise.  Use the following prompts if needed:
   1. How do the 2 drawings compare?
   2. Did the ‘giver’ and the ‘receiver’ have the same goals/objectives?  Did that make it confusing? Frustrating?
   3. What were the difficulties in communication?  Why did these occur?
   4. How did the communication feel?
   5. How could you improve the interaction?
   6. Ask the group to discuss how this might relate this to the ED role- *ED physicians have frequent communication with those not on their primary team (specialists, consultants, attendings, nurses, etc).*
   7. Do consulting services always have the same understanding of the objectives regarding patient care as the primary team does?
   8. How does setting expectations up front change a conversation or interaction?
   9. How do you make sure you get the answer to the clinical question you’re asking your consultants?  *Think about early, clear communication of your objective.  Convey severity, time-frame if sensitive, relative importance.*
3. Review the crucial elements of a consult call.
   1. Can review Kessler’s 5 Cs concept as a guide
      1. *A teaching model for physician consultation was developed and validated by Chad Kessler. Originally adapted from a business model identifying the core components of consultation, called “Kessler’s 5C Consultation” model. The model breaks down five core components to consider when contacting a consultant, including: Contact, Communicate, Core question, Collaborate, and Close the loop*
   2. Review each of the 5 Cs and give examples of each.

Kessler’s 5 Cs:

Contact:

*Examples: “my name is Dr. X, I am a senior resident working in the emergency department. We have you listed as the on-call pediatric surgeon today, is that correct?”*

Communicate:

*Examples: This should be a concise story with pertinent information. “The patient presented to the ED with…labs and imaging have shown…so far, the patient has received…”*

Core Question:

*Examples: “I would like your recommendation on empiric antibiotics in this patient as I am concerned about X infection”*

Collaboration:

*Examples: This is a discussion between the consultant and emergency department provider and gives the consultant a chance to ask questions and provide initial input. “Do you have any questions about the patient?”*

Closing:

*Examples: This helps close the loop and ensure the consultant and emergency department provider are on the same page about next steps. “After your team has recommendations, please call me back at X number to discuss next steps”. This is also a time to thank the consultant*

- 1. Connect Kessler’s 5 Cs to the core consultation components reviewed during the clinical case activity

Connecting Kessler’s 5 Cs and the Core Consultation Components:

Contact:

1. Introduced themselves
2. Introduced their role
3. Introduced the setting
4. Confirmed correct consultant
5. Provided patient information

Core Question:

1. Provided a clear core question

Communicate:

1. Provided appropriate patient presentation
2. Provided appropriate labs
3. Provided appropriate imaging
4. Provided appropriate interventions

Collaboration:

1. Offered a chance to ask questions

Closing:

1. Communicated Plan regarding next steps
2. Said thank you
3. Make explicit connections between the art exercise and Kessler’s 5 Cs using a table or chart as a visual cue.

|  | **Art Exercise** | **Consult Call** |
| --- | --- | --- |
| **Contact** | Name, materials | Name, setting, MRN |
| **Communicate** | Big picture overview, then details | Clinical concern, then case details |
| **Core Question** | Clear “ask”: ‘we will be drawing a house | Clear “ask”: ‘I'm requesting…’ |
| **Collaboration** | Back and forth collaboration | Clarify questions and needs |
| **Closing** | ‘Do you have questions?’ | Time frame for consultation  ‘Do you have any questions?’ |

**Artistic Activity #2 (in pairs): (5 min)**

1. Participants A and B switch roles.
2. Repeat the exercise again with the train drawing
3. Partners should still not look at the other’s page, but can now give additional context or general instructions

**Group Rapid Debrief: (< 5 min)**

1. How do the 2 drawings compare?  Any improvement from the previous exercise?
2. What did you do differently this time around?
3. How did the communication feel?

**Clinical Case Activity #2 (in pairs): (5 min)**

1. Give Participant A the clinical case file again. Instruct Participant A to open the case file to their unused case (#1 or #2, whichever was not used initially)
2. Give Participant B a new scoring QR code or physical evaluation sheet, Appendix C.
3. Give all participants 30 seconds to look over their materials.
4. Participant A should perform a mock consult call where they are the ED physician calling a consulting service (Participant B). Participant B may ask clarifying questions but is not required to. This should take less than 2 minutes. Cases are available in Appendix B.
5. Participant B should complete the evaluation sheet and submit it electronically or in person.

**Feedback: (1 min):**

Have all participants fill out a 1- minute feedback survey regarding the quality and content of the session prior to leaving the venue, Appendix E.

**Additional Resources:**

For facilitators wishing to review additional information regarding consultation calls and Kessler’s 5 C’s model, we suggest the following online resources and references:

- “How to Call a Consult,” REBEL EM blog by Rob Bryant. Mar 17, 2016. Available at: <https://rebelem.com/how-to-call-a-consult/>.
- “Requesting Consultations Using Kessler’s 5C’s,” CandadiEM blog post by Nadim Lalani. Mar 4, 2013. Available at: <https://canadiem.org/requesting-consultations-dr-rob-woods/>
- “Sweating Bullets and Killing ‘em with Kindness,” The Short Coat blog post by Lauren Westafer. Oct 10, 2012. Available at: <https://shortcoatsinem.blogspot.com/2012/10/sweating-bullets-and-killing-em-with.html>
- Kessler C et al. Consultation in the Emergency Department: A Qualitative Analysis and Review. J Emerg Med. 2012;42(6):704-711.
