# Supplementary material for: The Art of the Consult Call: Improving Communication Through Shared Mental Models
Source: MedEdPORTAL. 2023 Sep 29;19:11347. doi: 10.15766/mep_2374-8265.11347 (PMC10539490; doi:10.15766/mep_2374-8265.11347)
Supplement: Supplementary file 1 — Session Overview.docxConsultation Cases 1 and 2.docxEvaluation and Consultation Components.docxDrawing Activity Materials.docxCurriculum Feedback Survey.docx [file mep_2374-8265.11347-s001.zip › C. Evaluation and Consultation Components.docx]

**Anonymous Evaluation:**

*For all questions below, please provide information about the person who is acting as the ED physician during the simulated consult call.*

1. Please enter the initials of the person who will be acting as the ED physician (talking to you). *Please note: This is for reference only, all responses will remain anonymous.*_________
2. Please indicate the current level of training for the person acting as the ED physician.

MS3

MS4

PGY1

PGY2

PG3

Fellow

Faculty

1. Did the physician introduce themselves, their role, and their setting? (Ex: “my name is Dr X; I’m a senior resident working in the University ED”). Please check all that apply.

Introduced themselves

Introduced their role

Introduced their setting

None of the above

1. Did the physician confirm that they were talking to the correct consultant (ex: “are you the resident taking Pediatric Orthopedic consults today?”)

Yes

No

1. Did the physician provide or offer to provide identifying patient information (ex: “do you need the name or medical record number?”)

Yes

No

1. Did the physician verbalize a clear core question for the consultant? (ex: “I would like the pediatric surgery team to consult to help us decide if this patient requires admission to your service, antibiotics, and surgery for appendicitis.”)

Yes

No

1. Were appropriate details given about the patient’s presentation, including relevant labs, imaging, and interventions? (Ex: the patient presented to the ED with...labs and imaging have shown...so far, the patient has received....). please check all details that were given.

Presentation

Labs

Imaging

Interventions

None

1. Did the physician offer a chance for questions or clarifications before ending the call? (ex: “do you have any other questions I can answer?”)

Yes

No

1. Did the physician discuss the plan regarding next steps for communication about this patient? (ex: “after your team has recommendations, please stop by the desk or call me on the phone to discuss next steps”)

Yes

No

1. Did the physician thank you for taking the call or providing the consultation service? (Ex: “I appreciate you taking this call”)

Yes

No

**Core Consultation Components:**

1. Introduced themselves
2. Introduced their role
3. Introduced the setting
4. Confirmed correct consultant
5. Provided patient information
6. Provided a clear core question
7. Provided appropriate patient presentation
8. Provided appropriate labs
9. Provided appropriate imaging
10. Provided appropriate interventions
11. Offered a chance to ask questions
12. Communicated Plan regarding next steps
13. Said thank you
