# Supplementary material for: The Art of the Consult Call: Improving Communication Through Shared Mental Models
Source: MedEdPORTAL. 2023 Sep 29;19:11347. doi: 10.15766/mep_2374-8265.11347 (PMC10539490; doi:10.15766/mep_2374-8265.11347)
Supplement: Supplementary file 1 — Session Overview.docxConsultation Cases 1 and 2.docxEvaluation and Consultation Components.docxDrawing Activity Materials.docxCurriculum Feedback Survey.docx [file mep_2374-8265.11347-s001.zip › D. Drawing Activity Materials.docx]

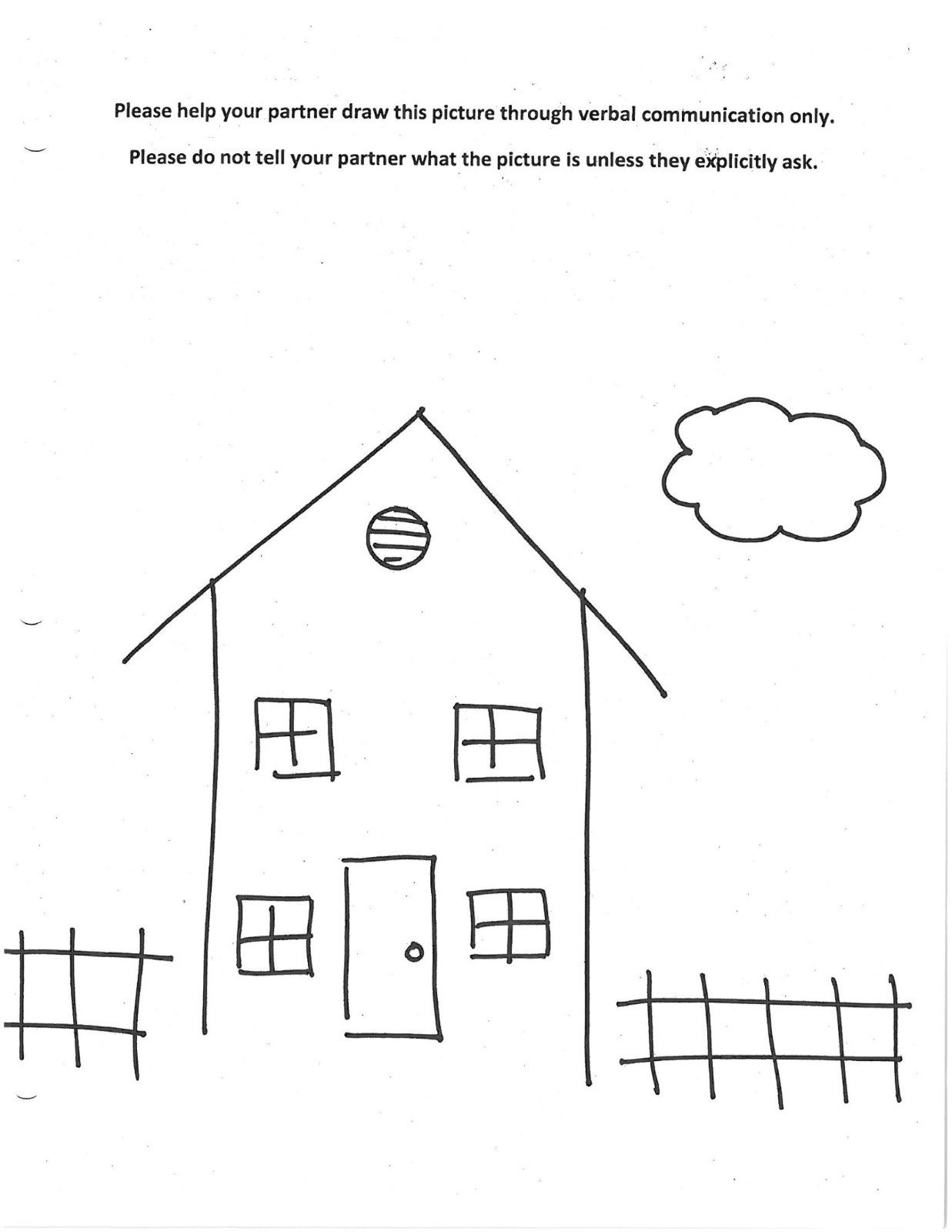


Author owned

Your partner will be helping you to draw a boat.

Please draw your picture in the space below.


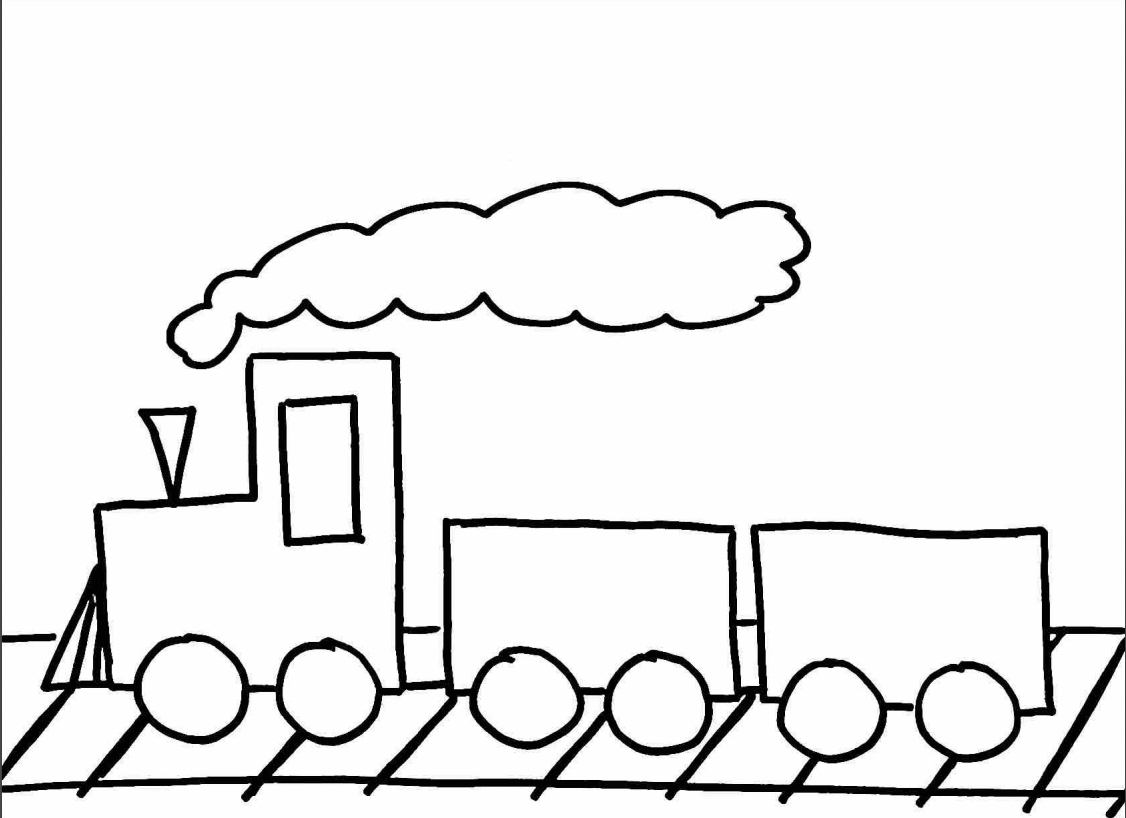

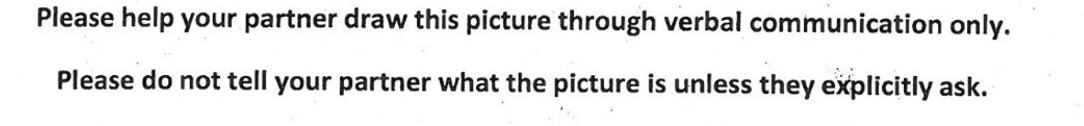


Author owned

Your partner will be helping you to draw a picture.

Please draw your picture in the space below.
