# Supplementary material for: The Art of the Consult Call: Improving Communication Through Shared Mental Models
Source: MedEdPORTAL. 2023 Sep 29;19:11347. doi: 10.15766/mep_2374-8265.11347 (PMC10539490; doi:10.15766/mep_2374-8265.11347)
Supplement: Supplementary file 1 — Session Overview.docxConsultation Cases 1 and 2.docxEvaluation and Consultation Components.docxDrawing Activity Materials.docxCurriculum Feedback Survey.docx [file mep_2374-8265.11347-s001.zip › E. Curriculum Feedback Survey.docx]

Curriculum Evaluation and Feedback Survey

1. Presentation was clear and organized

Strongly disagree Disagree Neutral Agree Strongly agree

1. Instructor was an effective communicator

Strongly disagree Disagree Neutral Agree Strongly agree

1. Instructor engaged the audience

Strongly disagree Disagree Neutral Agree Strongly agree

1. Instructor stimulated interest in the topic

Strongly disagree Disagree Neutral Agree Strongly agree

1. State objectives of the lecture were met

Strongly disagree Disagree Neutral Agree Strongly agree

1. Content was organized and well planned

Strongly disagree Disagree Neutral Agree Strongly agree

1. Content was appropriate for my level of training

Strongly disagree Disagree Neutral Agree Strongly agree

1. Lecture encouraged participants to participate

Strongly disagree Disagree Neutral Agree Strongly agree

1. Visual teaching aides (slides, videos) were well designed or displayed

Strongly disagree Disagree Neutral Agree Strongly agree

1. Will this lecture alter or improve your clinical practice?

Yes No Maybe

1. Would you recommend this lecture be repeated for other trainees?

Yes No Maybe

1. What aspects of this lecture were most useful or valuable?
2. How would you improve this lecture?
