# Supplementary material for: The Art of the Consult Call: Improving Communication Through Shared Mental Models
Source: MedEdPORTAL. 2023 Sep 29;19:11347. doi: 10.15766/mep_2374-8265.11347 (PMC10539490; doi:10.15766/mep_2374-8265.11347)
Supplement: Supplementary file 1 — Session Overview.docxConsultation Cases 1 and 2.docxEvaluation and Consultation Components.docxDrawing Activity Materials.docxCurriculum Feedback Survey.docx [file mep_2374-8265.11347-s001.zip › B. Consultation Cases 1 and 2.docx]

**Case #1: Neurology Consult**

Cc: Seizure

HPI: 11-month-old here with concern for seizure activity

Coming in with the second episode of possible seizure activity. 1^st^ one (described below) happened ~15 hours ago. She was seen at an outside emergency department and was well appearing so discharged home with plan for outpatient EEG + neuro

Mom witnessed the events:

1^st^ event:

- Infant was playing on the ground when she fell back and started moving all of her limbs, like shaking/jerking. Her eyes also rolled in the back of her head
- Lasted about 2 minutes
- Afterward seemed sleepyfor about an hour

2^nd^ event happened just prior to arrival

- This time her left arm was moving first, and her eyes were stuck to one side (mom can’t remember which way she was looking)
- After about a minute the rest of her limbs started shaking just like before
- Total lasted about 4 minutes
- Seemed sleepy afterward but mom says she started acting more like herself after about 30 minutes

Patient has been afebrile. A slight runny nose for the past week but not worsening. No cough. Tolerating PO intake. Normal UOP. No vomiting or diarrhea. No rashes.

Nothing like this has ever happened before

No trauma

PMHx:

Born term, uncomplicated pregnancy and delivery

No hospitalizations or surgeries, UTD on immunizations

No medications or allergies

Family history: (you forgot to ask about family history of seizures)

VS: T 37.5, HR 146, RR 22, BP 115/67, 99% on RA

Exam: playful, awake, alert, interactive

HEENT: TMs clear, MMM, no cervical lymphadenopathy

Cardio: RRR with no murmurs, rubs, gallops. Cap refill < 2 seconds

Pulm: clear bilaterally, no increased work of breathing, no crackles/wheezes/rhonchi

Abd: soft, non-tender, non-distended, no masses

Extremities: warm and well perfused

Skin: no rashes or lesions

Neuro: awake, playful, interactive with mom. PERRL. Face symmetric movements. Moving all extremities equally and spontaneously. Pulls to stand with mom.

Labs:

Capillary blood glucose of 82mg/dl

Imaging: none obtained

PLAN:

- Talk to neuro given this is the second episode and maybe focal this time....

**Case #2: pediatric general surgery consult**

Cc: abdominal pain

HPI: 7yo M with acute RLQ abdominal pain and vomiting

Symptom onset 3 days ago. Pain was initially periumbilical but now more RLQ

Has not wanted to eat or drink much

A few episodes of vomiting each day

Fevers: Tmax 101 today

UOP: normal

BMs: always somewhat constipated, last BM the day before symptoms started

No respiratory symptoms such as cough, runny nose, sore throat

No sick contacts

PMHx:

Otherwise healthy

No surgeries

UTD on immunizations

No medications or allergies

Unremarkable family history

VS: T 38.2, HR 129, BP 102/71, RR 21, Sats 98% RA

Gen: awake, alert, mildly anxious appearing but distractable

HEENT: TMs clear, MMM, no cervical lymphadenopathy

Cardio: RRR with no murmurs, rubs, gallops. Cap refill < 2 seconds

Pulm: clear bilaterally, no increased work of breathing, no crackles/wheezes/rhonchi

Abd: soft. + tenderness to palpation RLQ with some voluntary guarding but no rebound

Extremities: warm and well perfused

Skin: no rashes or lesions

Neuro: awake, playful. PERRL. Moving all extremities equally and spontaneously.

Labs:

White blood cell count: 13 K/cu mm

Hemoglobin: 12.5 g/dL

Platelets: 238 K/cu mm

C-reactive protein: 15 mg/L (reference range <10.0)

Na 138 mmol/L

K 3.4 mmol/L

Cl 98 mmol/L

CO2 21 mmol/L

BUN 12 mg/dL

Cr 0.68 mg/dL

US: unable to visualize appendix

No free fluid, fat stranding, or lymph nodes

Meds:

20ml/kg NS bolus

Tylenol

Morphine x 1

PLAN:

- talk to surgery as the patient is still painful on examination (the morphine did help briefly and he was able to comfortably walk to the bathroom)
