# Supplementary material for: The Art of the Consult Call: Improving Communication Through Shared Mental Models
Source: MedEdPORTAL. 2023 Sep 29;19:11347. doi: 10.15766/mep_2374-8265.11347 (PMC10539490; doi:10.15766/mep_2374-8265.11347)
Supplement: Supplementary file 1 — Session Overview.docxConsultation Cases 1 and 2.docxEvaluation and Consultation Components.docxDrawing Activity Materials.docxCurriculum Feedback Survey.docx [file mep_2374-8265.11347-s001.zip › _Educational Summary Report_11347.pdf]

MedEdPORTAL® The AAMC Journal of Teaching and Learning Resources

Original Publication 	Open Access

The Art of the Consult Call: Improving Communication Through Shared Mental Models

Monica Saladik, MD*, Michelle Noelck, MD, Jessica Bailey, MD

*Corresponding author: saladik@ohsu.edu

Abstract

Introduction: The Accreditation Council for Graduate Medical Education cites effective communication with physicians as a core competency for emergency medicine (EM) residents. However, there is no standardized curriculum dedicated to communication beyond practice in the clinical setting. **Methods:** We developed a 1-hour EM didactic session on effective consultations using experiential education principles. Learners were placed in pairs of one junior learner and one senior learner. The junior learner performed a mock phone consultation using an EM patient case; the senior learner completed an online evaluation, assessing the junior learner on 13 core components of a successful consult call, based on Kessler's 5Cs consultation model. Subsequently, learners participated in an intervention, which included an artistic activity and facilitated debrief, connecting their reflections to clinical practice. Postintervention, the same paired learners completed a second mock consultation call and reevaluation. Finally, learners completed a feedback survey. **Results:** Fifteen pairs completed both the pre- and postintervention evaluations. Of the junior learners simulating the consultation call, 47% were clinical medical students, and 53% were first-year EM residents. Preintervention, learners completed a mean of 51% of core consult call components compared to a mean of 84% postintervention. This 33% improvement was statistically significant ($p < .001$; 95% CI, 19.9-46.1). Eight participants completed the feedback survey; 100% agreed or strongly agreed with positive statements regarding overall session content. **Discussion:** This engaging interactive session utilizing a mock communication exercise, unique artistic activity, and guided reflection can effectively increase junior learners' phone consultation communication skills.

Keywords
Consultation Call, Experiential Education, Mock Phone Consultation, Communication Skills, Emergency Medicine, Pediatric Emergency Medicine, Simulation

Educational Objectives

By the end of this session, learners will be able to:

1. Summarize the elements of Kessler's 5 Cs of a consult call.
2. Connect the communication skills required for effective paired drawing to the elements of consultation.
3. Apply the elements of Kessler's 5 Cs in mock communication exercises.

Introduction

Physicians commonly discuss patient cases with consultants. This practice is often conducted via the phone in an emergency department (ED) setting, with an estimated 20%-40% of patients in EDs requiring at least one consultation during their visit.[1] The Accreditation Council for Graduate Medical Education (ACGME) cites effective communication with physicians as a core competency for emergency medicine (EM) residents.[2] However, there is a lack of standardization in formal curricula regarding physician-to-physician communication and consultation.[3] Additionally, survey data suggest that the majority of physicians feel medical students and residents are inadequately trained in consultation skills.[4]

A teaching model for physician consultation has been developed and validated by Chad Kessler.[3-5] This model was originally adapted from a business model identifying the core components of consultation, called Kessler's 5Cs consultation model.[3] The model describes five core components to consider when contacting a consultant, including contact, communicate, core question, collaborate, and close the loop.[6] Each of the Cs also has a specific checklist item associated with it.[5,6] While Kessler's model has been studied prospectively with medical students

Citation:
Saladik M, Noelck M, Bailey J. The art of the consult call: improving communication through shared mental models. *MedEdPORTAL*. 2023;19:11347. https://doi.org/10.15766/mep_2374-8265.11347

Copyright © 2023 Saladik et al. This is an open-access publication distributed under the terms of the Creative Commons Attribution license.

using both live and asynchronous interventions, no standardized didactic content beyond the checklist itself exists that can be easily added to a residency or clinical medical student curriculum.[3]

Currently, only limited resources specific to this topic have been published. There are two separate 4-hour sessions for medical students transitioning to internship, both of which have a portion dedicated to obtaining a consultation.[7,8] However, they are relatively lengthy to incorporate into an established longitudinal residency curriculum and are not solely dedicated to the topic of consultation. An entrustable professional activity (EPA) has also been developed to provide interns with feedback on performing consultations.[9] This EPA was developed based on guidelines different from Kessler's model. Additionally, the EPA was designed for inpatient consultation and requires integration into clinical rotations over time, rather than being a discrete didactic session for all trainees at once.

Our goal was to create a short session on physician consultation and communication based on a validated model and interactive with multiple adult learning modalities. The curriculum was designed using principles of experiential education, a teaching philosophy that pairs creative hands-on experiences with facilitated debriefs to help learners apply abstract ideas to real-life situations.[10] Specifically, we used the approach of a four-phase Kolb cycle, in which an educator guides participants through a concrete experience, followed by periods of reflective observation and abstract conceptualization and finally an opportunity to experiment by applying their theories to a new situation.[11] We chose an artistic activity as the interactive component as it was novel to our participants and some prior data have shown art-based medical sessions are useful in skills such as physician communication.[12]

We developed the session content using Kessler's validated 5Cs consultation model. The 1-hour time frame allowed the curriculum to be easily added to EM residency didactic sessions. The session included both clinical medical students and resident learners, increasing its applicability across the spectrum from undergraduate- to graduate-level medical education.

Methods

We developed this session as a component of our institution's weekly EM residency required didactic curriculum. EM residents were the primary target learners, but medical students who were on a clinical rotation in the ED also participated. We developed the session to be 1 hour in length and divided it into three sections: the preintervention simulated consultation call, the intervention itself, and a postintervention simulated consultation call. An hour was adequate time to complete all activities, though the session could also be comfortably extended to 90 minutes. There was no formal prebriefing, but a basic session outline was reviewed with learners prior to starting. No prework was assigned to the learners. The single 1-hour session was led by a fellow and attending-level physician, though it could also be led by a senior resident.

Educational Session

We provided facilitators with a handout including a list of all required materials and a description of the session flow (Appendix A).

The session started with a clinical case activity in which learners were placed in pairs of one junior learner and one senior learner. Specifically, second- and third-year residents were asked to pair with either a medical student or intern. The junior learner was given time to briefly read through a pediatric EM case requiring phone consultation with a subspecialty service. Both this case and the postintervention case (Appendix B) were created by a pediatric EM fellow based on similar real-life experiences and reviewed by the attending fellowship director. The junior learner simulated a consultation call to the senior learner, using written case 1 as a reference. The senior learner filled out an anonymous online evaluation (Appendix C) assessing whether the junior learner had completed each of the 13 core components of the consultation call. These components were modeled after Kessler's 5Cs consultation model[3-5] and included such questions as these: Did the physician introduce themselves? Did the physician verbalize a clear core question? Did the physician offer a chance for questions or clarifications before ending the call?

The intervention itself included a paired artistic activity (Appendix D), followed by a debrief and guided didactic connecting it to the clinical concept. The learners were kept in their original pairing and instructed to sit back-to-back so they could not see their partner's paper. The junior learner was given a sheet that contained a simple drawing of a house and yard and had the instructions "Please help your partner draw this picture through verbal communication only. Please do not tell your partner what the picture is unless they explicitly ask." The senior learner was given a blank sheet of paper with the instructions "Your partner will be helping you to draw a boat. Please draw your picture in the space below." After learners were given time to complete the activity, there was a facilitator-guided debrief session. This session was based on the experiential education format: Learners were encouraged to reflect on the activity, unexpected difficulties that arose, and how communication might

Copyright © 2023 Saladik et al. This is an open-access publication distributed under the terms of the Creative Commons Attribution license.

have been improved. They were also encouraged to discuss how these observations might translate to their roles in the ED. After the debrief, the facilitators introduced and reviewed the core elements of the consultation call, using Kessler's 5Cs consultation model as a guide.

The learners subsequently completed the same artistic activity (Appendix D). The junior learner was given a sheet that contained a simple drawing of a train and had the instructions "Please help your partner draw this picture through verbal communication only." The senior learner was given a blank sheet of paper with the instructions "Your partner will be helping you to draw a picture. Please draw your picture in the space below." This was followed by a rapid debrief and reflection on how communication was different the second time around.

After the intervention, the same learner pairs reconvened. The junior learner was given a second pediatric EM case and simulated a consultation call to the senior learner, using written case 2 (Appendix B) as a reference. The senior learner filled out the same anonymous online evaluation, assessing whether the junior learner had completed each of the same 13 core components for this second consultation call.

Curriculum Evaluation
At the end of the session, all learners were given an opportunity to complete an anonymous online feedback survey (Appendix E) subjectively evaluating the session itself. The survey included questions regarding the effectiveness of the instructors, the lecture content, and whether the respondent would recommend the session in the future. Also, the pre- and postintervention evaluation forms (Appendix C) were compared to objectively assess whether the intervention had an immediate impact on learners successfully delivering all the core components of a consultation call.

Results

A total of 33 learners participated in the didactic session. Fifteen learner pairs completed all components of the session. Forty-seven percent of the junior leaders who simulated the consultation call were either third- or fourth-year clinical medical students, and 53% were first-year EM residents. Preintervention evaluation results indicated that learners initially completed 51% of the core components of the consult call. Postintervention evaluation results indicated that learners completed 84% of core components of the consult call. The mean difference of 33% improvement between pre- and postintervention was statistically significant ($p < .001$; 95% CI, 19.9-46.1; Figure 1).

Figure 2 shows the breakdown percentage of learners who reported each component individually for both pre- and postintervention.

Eight learners completed the session feedback survey. All respondents agreed or strongly agreed with the 5-point Likert scale questions (1 = *strongly disagree,* 5 = *strongly agree*) regarding the skills and responsiveness of the instructor and the lecture content, including that the presentation was clear and organized, the stated objectives were met, and the lecture encouraged participation. One learner was neutral regarding the statement that the visual aids were well designed or displayed,

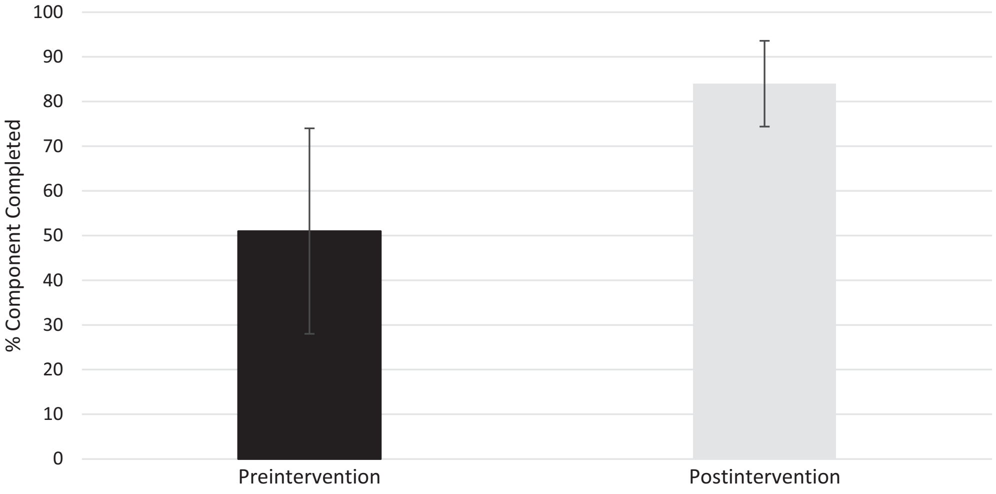

Figure 1. Overall core components completed by the junior learners both preintervention (51%) and postintervention (84%). Error bars demonstrate that the mean difference of 33% improvement between the pre- and postintervention was statistically significant ($p < .001$; 95% CI, 19.9-46.1).

Copyright © 2023 Saladik et al. This is an open-access publication distributed under the terms of the Creative Commons Attribution license.

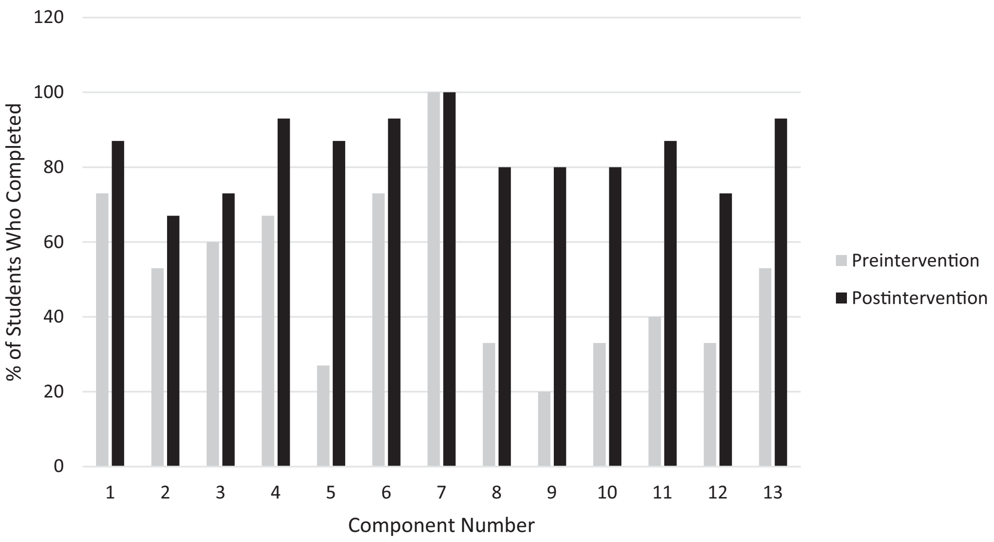

Figure 2. Individual breakdown percentage of learners who completed each component during the pre- and postintervention. Appendix B provides a full description of each component assessed on the survey.

while the remaining learners either agreed or strongly agreed. The questions and learner responses are shown in Figure 3.

All eight respondents (100%) indicated that the lecture would alter or improve their clinical practice and that they would recommend the lecture be repeated for other trainees.

Discussion

This 1-hour interactive didactic session was developed using a validated teaching model for effective consultation paired with a unique art-based experiential educational format. The session was designed to evaluate both Kirkpatrick level 1 (learners' reaction to the educational intervention) and level 2 (influence on learners' behavior).[13] The results of the pre- and postintervention evaluations demonstrated a statistically significant increase in junior learners' ability to effectively communicate during a consultation call as perceived by senior learner evaluators. Additionally, feedback indicated that all participants felt the session would improve clinical practice and recommended it be repeated for other trainees. This session can be used as a stand-alone offering or as part of a larger communication series. The session is also suitable for both clinical medical students and junior resident learners. The facilitators agreed that our methods and design provided a smooth educational session that was engaging for learners. A possible modification would be to allow the session to be conducted over 1.5 hours instead of 1 hour. Another possible modification would be to eliminate the second artistic activity to help ensure all groups of learners have enough time for robust debriefing.

Our curricular session does have limitations. First, the small number of participants and the focus on EM learners in the ED setting could limit its overall generalizability to other subspecialties. Similarly, as this was a single-center project, there is some limitation on extrapolation to other centers that might have a different culture of consultation. The session builds on the assumption that the presence of Kessler's core components in a consultation call does in fact improve the quality and effectiveness of the call. One prospective study assessing senior medical students with a standardized phone consultation call did not demonstrate an improvement in effective communication based on physician consultant review of the phone call.[14] However, data for the development of Kessler's model were based on both surveys of EM and consultant physicians and subsequent validity evidence. Thus, that prospective study highlights the variability regarding physician perception of effective communication but does not invalidate standardized approaches to communication curricula. Lastly, our project did not address the possibility of skill decay, as the pre- and postintervention evaluations were done immediately after the teaching intervention.

Despite these limitations, we have demonstrated that this 1-hour session can be an effective way to increase learners' ability to deliver more structured and complete consultation calls. The use of a validated model for consultation calls does provide a method for standardizing a complex communication skill. Recent literature highlights the idea that residency curricula, specifically

Copyright © 2023 Saladik et al. This is an open-access publication distributed under the terms of the Creative Commons Attribution license.

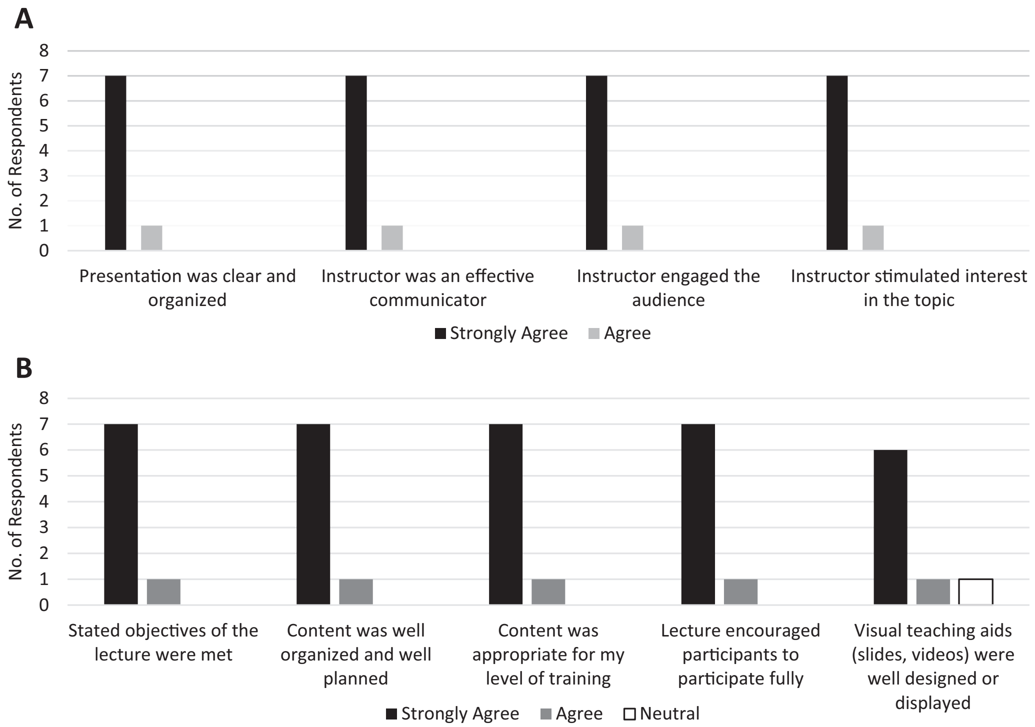

Figure 3. List of questions and learner responses from the postsession feedback survey. A: Skill and responsiveness of instructor. B: Lecture content. Responses were based on a 5-point Likert scale (1 = *strongly disagree,* 5 = *strongly agree*). No participants chose the *disagree* or *strongly disagree* options on any survey question assessed.

those for EM programs, should decrease reliance on traditional lecture-based didactics and aim to activate learners through a range of immersive formats.[15,16] Utilizing experiential education theory to build hands-on didactics aligns with the ACGME's requirements for programs to provide varied instructional methods.[2] Several studies have shown that art-based medical education sessions can help with physician communication and a variety of other skills such as observational skills and increased empathy.[12,17] Thus, the addition of this interactive session to the curriculum provides a valuable, creative, and engaging component and, thus, a multimodal strategy for increasing effective communication.

Planned future directions for this work include follow-up surveys at 6-month intervals to determine whether learners perceive retention of their consultation skills. Future considerations also include faculty assessment of residents who have completed this session to assess for skill decay. Additionally, it would be beneficial to expand assessment of effectiveness in the workplace (reaching Kirkpatrick level 3) by surveying faculty on whether learners who have participated in the session show more effective consultation skills than peers who have not completed it. Similarly, consultant or subspecialty faculty could be added to the session to rate communication, rather than relying solely on resident assessment. Lastly, with slight modifications, the session could easily be adapted for use in various residency training programs or to teach other types of health care providers, increasing its generalizability.

Appendices

A. Session Overview.docx

B. Consultation Cases 1 and 2.docx

C. Evaluation and Consultation Components.docx

D. Drawing Activity Materials.docx

E. Curriculum Feedback Survey.docx

All appendices are peer reviewed as integral parts of the Original Publication.

Monica Saladik, MD: Pediatric Emergency Medicine Fellow, Department of Emergency Medicine, Oregon Health & Science University School of Medicine; ORCID: https://orcid.org/0000-0002-9273-8189

Michelle Noelck, MD: Associate Professor, Department of Pediatrics, Oregon Health & Science University School of Medicine

Copyright © 2023 Saladik et al. This is an open-access publication distributed under the terms of the Creative Commons Attribution license.

Jessica Bailey, MD: Assistant Professor, Department of Emergency Medicine, Oregon Health & Science University School of Medicine

Disclosures
None to report.

Funding/Support
None to report.

Ethical Approval
Reported as not applicable.

References

1. Lee RS, Woods R, Bullard M, Holroyd BR, Rowe BH. Consultations in the emergency department: a systematic review of the literature. *Emerg Med J*. 2008;25(1):4-9. https://doi.org/10.1136/emj.2007.051631
2. *ACGME Program Requirements for Graduate Medical Education in Emergency Medicine*. Accreditation Council of Graduate Medical Education; 2022. Accessed August 8, 2023. https://www.acgme.org/globalassets/pfassets/programrequirements/110_emergencymedicine_2022_tcc.pdf
3. Kessler CS, Tadisina KK, Saks M, et al. The 5Cs of consultation: training medical students to communicate effectively in the emergency department. *J Emerg Med*. 2015;49(5):713-721. https://doi.org/10.1016/j.jemermed.2015.05.012
4. Kessler C, Kutka BM, Badillo C. Consultation in the emergency department: a qualitative analysis and review. *J Emerg Med*. 2012;42(6):704-711. https://doi.org/10.1016/j.jemermed.2011.01.025
5. Kessler CS, Kalapurayil PS, Yudkowsky R, Schwartz A. Validity evidence for a new checklist evaluating consultations, the 5Cs model. *Acad Med*. 2012;87(10):1408-1412. https://doi.org/10.1097/ACM.0b013e3182677944
6. Lalani N. Requesting consultations using Kessler's 5-Cs. CanadiEM. March 4, 2013. Accessed August 8, 2023. https://canadiem.org/requesting-consultations-dr-rob-woods/
7. Burns R, Mangold K, Adler M, Trainor J. Pediatric Boot Camp series: obtaining a consult, discussing difficult news. *MedEdPORTAL*. 2016;12:10437. https://doi.org/10.15766/mep_2374-8265.10437
8. Tischendorf J, O'Connor C, Alvarez M, Johnson S. Mock paging and consult curriculum to prepare fourth-year medical students for medical internship. *MedEdPORTAL*. 2018;14:10708. https://doi.org/10.15766/mep_2374-8265.10708
9. Kang AJ, Gielissen K, Windish D. Using an Entrustable Professional Activity to assess consultation requests called on an internal medicine teaching service. *MedEdPORTAL*. 2019;15:10854. https://doi.org/10.15766/mep_2374-8265.10854
10. What is experiential education? Association for Experiential Education. Accessed August 8, 2023. https://www.aee.org/what-is-experiential-education
11. Kolb DA. *Experiential Learning: Experience as the Source of Learning and Development*. Prentice-Hall; 1984.
12. Yakhforoshha A, Emami SAH, Shahi F, et al. Effectiveness of integrating simulation with art-based teaching strategies on oncology fellows' performance regarding breaking bad news. *J Cancer Educ*. 2019;34(3):463-471. https://doi.org/10.1007/s13187-018-1324-x
13. Kirkpatrick JD, Kirkpatrick WK. *Kirkpatrick's Four Levels of Training Evaluation*. ATD Press; 2016.
14. Turner J, Litzau M, Morgan ZS, Pollard K, Cooper DD. Failure of an educational intervention to improve consultation and implications for healthcare consultation. *Cureus*. 2019;11(2):e4105. https://doi.org/10.7759/cureus.4105
15. Wray A, Wolff M, Boysen-Osborn M, et al. Not another boring resident didactic conference. *AEM Educ Train*. 2019;4(suppl 1):S113-S121. https://doi.org/10.1002/aet2.10367
16. Toohey SL, Wray A, Wiechmann W, Lin M, Boysen-Osborn M. Ten tips for engaging the millennial learner and moving an emergency medicine residency curriculum into the 21st century. *West J Emerg Med*. 2016;17(3):337-343. https://doi.org/10.5811/westjem.2016.3.29863
17. Shapiro J, Rucker L, Beck J. Training the clinical eye and mind: using the arts to develop medical students' observational and pattern recognition skills. *Med Educ*. 2006;40(3):263-268. https://doi.org/10.1111/j.1365-2929.2006.02389.x

Received: December 13, 2022
Accepted: June 13, 2023
Published: September 29, 2023

Copyright © 2023 Saladik et al. This is an open-access publication distributed under the terms of the Creative Commons Attribution license.
